# Effect of Drugs on Lactation

**Published:** 1882-03

**Authors:** 


### EFFECT OF DRUGS ON LACTATION.

The practical conclusions of Dolan and Wood, in *Practitioner*, are: 1. Therapeutical agents intended to act on the mammary gland must enter the blood. 2. Drugs derived from the natural orders Liliaceæ, Cruciferæ, Solanaceæ, Umbelliferæ, etc., enter the blood and impregnate the milk, hence caution is needed in giving such drugs to nursing women. 3. The only approach to a true galactagogue is jaborandi. 4. Belladonna is an antigalactagogue. 5. In inaction of the mammæ the milk may be increased and influenced by medicines. 6. The milk may be increased in heat-forming elements by administration of fats. 7. The salts of milk are improved by administration of medicines. 8. Various physiological actions—purgative, alterative, diuretic, etc.—are produced in the child by giving drugs to the mother. 9. We must look to diet for improvement in milk-secreting power, both as to the quantity and quality of the milk.—*Louisville Med. News.*

